# RNN-Based Sequence to Sequence Decoder for Run-Length Limited Codes in Visible Light Communication

**DOI:** 10.3390/s22134843

**Published:** 2022-06-27

**Authors:** Xu Luo, Haifen Yang

**Affiliations:** Department of Information and Communication Engineering, University of Electronic Science and Technology of China, Chengdu 611731, China; fzu_luoxu@163.com

**Keywords:** run-length limited codes, seq2seq model, RNN-based decoding, unmanned aerial vehicles, visible light communication

## Abstract

Unmanned aerial vehicles (UAVs) equipped with visible light communication (VLC) technology can simultaneously offer flexible communications and illumination to service ground users. Since a poor UAV working environment increases interference sent to the VLC link, there is a pressing need to further ensure reliable data communications. Run-length limited (RLL) codes are commonly utilized to ensure reliable data transmission and flicker-free perception in VLC technology. Conventional RLL decoding methods depend upon look-up tables, which can be prone to erroneous transmissions. This paper proposes a novel recurrent neural network (RNN)-based decoder for RLL codes that uses sequence to sequence (seq2seq) models. With a well-trained model, the decoder has a significant performance advantage over the look-up table method, and it can approach the bit error rate of maximum a posteriori (MAP) criterion-based decoding. Moreover, the decoder is use to deal with multiple frames simultaneously, such that the totality of RLL-coded frames can be decoded by only one-shot decoding within one time slot, which is able to enhance the system throughput. This shows our decoder’s great potential for practical UAV applications with VLC technology.

## 1. Introduction

Visible light communication (VLC) [1,2,3], a typical short-range optical wireless communication using 380 to 780 nm in the visible light spectrum, has recently aroused much interest. In VLC, data transmissions are dependent on light sources with nanosecond switching times, such as light emitting diodes (LEDs) [4], which makes it highly suitable for search and rescue scenarios that require both illumination and communication. Reference [5] proposed the concept of “Twinkle”, which is based on the idea of using unmanned aerial vehicles (UAVs) equipped with LEDs for lighting applications such as urban security and disaster recovery. However, the severe communication environment of the UAVs, such as additional ambient illumination, may not ensure reliable data transmission [6]. To tackle this issue, error correction codes (ECCs) and RLL codes are considered. Additionally, RLL codes can achieve 50% dimming and flicker-free perception by guaranteeing equal numbers of 1 s and 0 s in RLL-coded sequences. Thus, RLL codes feature prominently in UAV communication systems equipped with VLC capabilities.

Deep learning (DL) techniques have achieved great success and have been employed in many areas of communication recently [7,8]. However, the key challenge for DL-aided channel decoding is the explosive number of training sets, as the length of the source word grows. For instance, with a BCH (63, 45) code, the training set contains up to $2^{45}$ different codewords, which is unpractical for training and establishing a DL-aided decoder to learn to recover transmitted information from a variety of noisy received signals [8,9,10]. Therefore, many papers have emerged to address the issue. One study [8] found that compared with random codes, structured codes, such as polar codes, are indeed easier to learn the codewords from that have never been seen during training. Another study [10] exploited DL techniques to optimize well-known decoding schemes and proposed a DL-aided belief propagation (BP) decoder. They employed non-fully connected neural networks to simulate the regular BP decoding. Since the performance of the BP algorithm is independent of the transmitted codewords, only a single codeword (e.g., the all-zero codeword) needs to be used for training. Realizing that BP decoding contains many multiplications, reference [11] improved the neural network (NN) architecture and achieved similar performance to [10] with lower complexity based on offset min-sum algorithm. As we will show in the rest of our paper, since the used RLL source words are normally fairly short, probably a few tens of bits at most [1,12,13], DL techniques fit well with RLL decoding, naturally avoiding the explosive numbers of training set data and weights that occur in ECC decoding. Thus, there is a pressing need to exploit DL techniques for RLL decoding in UAVs with VLC.

Recurrent neural networks (RNNs) are suitable for processing one-dimensional signals, especially sequence-based tasks with time dependence. Thus, this paper proposes an RNN-based decoder to improve the performance of RLL codes. We use a sequence to sequence (seq2seq) model with multiple-to-multiple configure (multiple inputs and multiple outputs) to learn the mappings between RLL source words and noisy received signals. We systematically analyze and demonstrate the effectiveness of our proposed decoder for RLL decoding. Uur contributions are as follows:

• We exploited RNN technology for RLL decoding based on seq2seq models and found that a well-trained seq2seq model can achieve MAP-based decoding performance, thereby improving the reliability of VLC.
• We show that the proposed decoder has sufficient capacity to handle multiple RLL-coded frames and implement multi-frame parallel decoding with only one-shot decoding, which largely improves the system throughput and reduces parallel decoding complexity.
• We found that the number of frames for parallel decoding impacts the fitting of the decoder due to its restricted learning abilities, and further analyzed the *saturation frame* of the decoder based on our proposed criterion.

The remainder of the paper proceeds as follows. Section 2 reviews the model of VLC and the RLL codes. Section 3 proposes the seq2seq decoder, including its design and the network training approach. Section 4 presents our simulation results, and Section 5 concludes the paper.

## 2. Related Background

### 2.1. The Commucation System Model of UAVs with VLC

**Figure 1:**
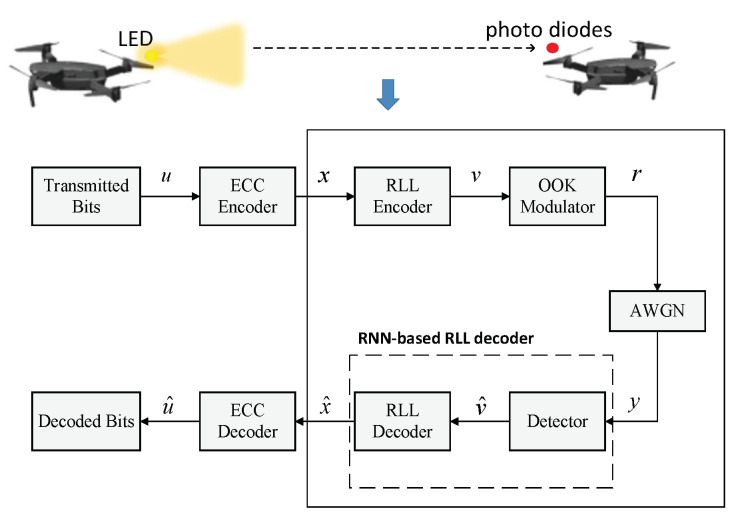
The communication system model of UAVs with VLC.

UAVs with VLC technology are used in a wide range of applications, such as indoor positioning and rescue scenarios [14]. To achieve positioning or distance measurement, UAVs exploit flashing LEDs to send useful information, such as their own coordinates, to the receiver, which can be any physical device with a photo diode, such as another UAV. After receiving the information, the receiver will perform further inference work [15]. Such a communication system can be abstracted as shown in Figure 1 [13,16,17]. Transmitted binary data u are encoded by the ECC encoder and RLL encoder to generate the encoded frames x and v, respectively. Later, the encoded frames are modulated by an OOK modulator into the electrical signal r, which is employed to drive the LEDs for lighting and transmission. After that, the modulated sequences are transmitted over an additive white Gaussian noise (AWGN) channel; hence, the received signal y is denoted by

(1) y=r+n,

where n follows $N\left(0,\sigma^{2}\right)$. The receiver consists of a detector, an RLL decoder, and an ECC decoder. The detector outputs the hard estimates by photo diodes (PDs), which are decoded by the RLL decoder and ECC decoder successively to obtain the transmitted data estimates.

### 2.2. RLL Codes

Since a poor UAV working environment, such as one with strong ambient illumination [6], increases the interference in the VLC link, there is a pressing need to ensure reliable data communications. In UAVs with VLC systems, RLL codes are usually used to ensure reliable data transmission and flicker-free perception. RLL encoder receives random binary data and divides them into length-*K* frames, and then maps each frame into length-*N* frames. Additionally, RLL codes can guarantee equal numbers of 1 s and 0 s in every RLL-coded frame for dimming adjustments and flicker-free perception. Conventional RLL decoder relies on table look-up decoding. It maps the hard decision bits $\hat{v}$ into a source word to recover the transmitted data. If the RLL decoder is not able to find the corresponding codeword in the codebook due to erroneous detection, the decoder will select the codeword with the minimum Hamming distance to $\hat{v}$. Three main types of RLL codes, namely, Manchester codes, 4B6B codes, and 8B10B codes, are commonly adopted in VLC systems. In 4B6B codes, there are 16 codewords, and each length-4 source word is mapped to a length-6 codeword, which is shown in Table 1 [4].

This paper establishes an RNN-based decoder to replace the “detector” and the “RLL decoder” in Figure 1, so that $\hat{x}$ is as close as possible to x. In our work, we ignore the effect of ECCs and consider 4B6B codes as an example. It should be emphasized that our work is also applicable to other RLL codes.

## 3. Learning to RLL Decode

### 3.1. Problem Formulation

We will consider the decoding of RLL codes as a DL problem. The inputs to the NN are the received signals $y=\left\{y_{1},y_{2},\ldots ,y_{N}\right\}$, where $y_{i}$ is the *i*-th symbol of the received signal and *N* is the codeword length. The outputs are a group of soft estimates $\tilde{x}=\left\{{\tilde{x}}_{1},{\tilde{x}}_{2},\ldots ,{\tilde{x}}_{K}\right\}$, corresponding to the transmitted data x, where *K* is the length of source word. Thereafter, we can obtain the final decoding results $\hat{x}=\left\{{\hat{x}}_{1},{\hat{x}}_{2},\ldots ,{\hat{x}}_{K}\right\}$ based on the soft estimates $\tilde{x}$ by the corresponding hard-decision rule:

(2) $$\left\{\begin{array}{c} {\hat{x}}_{i}=1,\;\mathrm{if}\;{\tilde{x}}_{i}>0.5\\ {\hat{x}}_{i}=0,\;\mathrm{if}\;{\tilde{x}}_{i}\leq 0.5 \end{array}\right..$$

We note that an input–output mapping of the NN is a chain function given by

(3) $$\tilde{x}=W\left(y;\theta \right)=W^{\left(L-1\right)}\left(W^{\left(L-2\right)}\left(\ldots \left(W^{\left(0\right)}\left(y\right)\right)\right)\right),$$

where *L* is the number of the NN layers and θ represents the network parameters. The NN will be trained to search the optimal $\theta^{*}$ by minimizing a properly defined the loss function L, given by

(4) $$\theta^{*}=\arg\min_{\theta }L\left(x,\tilde{x}\right),$$

where $L(x,\tilde{x})$ calculates the loss between $\tilde{x}$ and x. Our goal is to train the NN to find an optimal mapping function $W^{*}:y\to x$ which satisfies the MAP criterion:

(5) $$W^{*}\left(y;\theta^{*}\right)=\underset{x}{\arg\max}P\left(x|y\right).$$

Note that the training process is to be carried out offline. After the training and validation, the NN with the optimal $\theta^{*}$ and $W^{*}$ can then be embedded into UAVs to decode RLL codes.

### 3.2. Seq2seq Decoder

**Figure 2:**
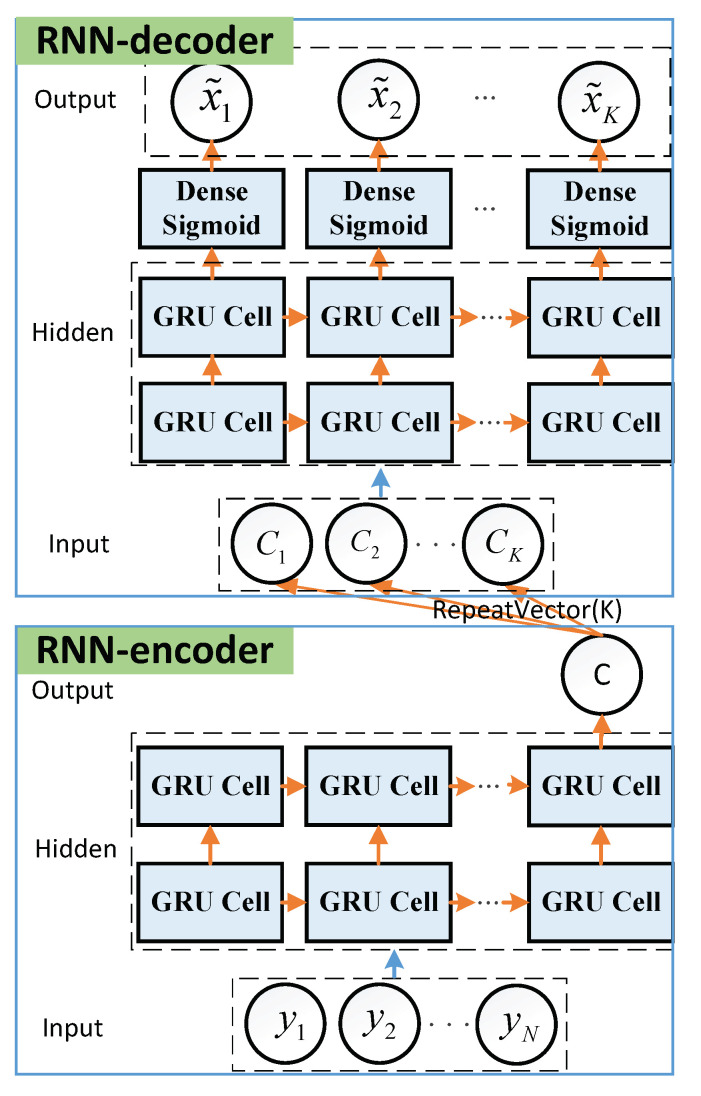
The seq2seq model for RLL decoding.

Analysis illustrates that the RLL decoding problem is the mapping of a received signal sequence to another bit sequence. It is available to be converted into a sequence-to-sequence problem with a many-input many-output structure. RNN has memories to process a sequence of inputs, and hence has strong capabilities for the time series tasks. A typical RNN-based model, namely, the seq2seq model, is suited to dealing with the sequence-based “*N* inputs and *K* outputs” tasks (N≠K) and learn mappings between unequal sequences due to its specific architecture [18,19]. Thus, we adopted the seq2seq model to perform our RLL decoding task. Figure 2 shows the model, which includes two RNN structures, i.e., RNN-encoder and RNN-decoder, respectively.

(1) *Training Phase:* In this phase, by training the adopted seq2seq decoder, we aim to make the output of the decoder close to the real decoded sequence. In the following, we introduce the input and output of the decoder, the loss function, and the training procedure.

- **Encoder-Input:** Denote $\left(y,x\right)$ as a training sample of dataset (y,x), with y being the the received signal and x being the corresponding label. Thus, the $y=\left\{y_{1},y_{2},\ldots ,y_{N}\right\}$ are fed into RNN-encoder as the inputs. The labels of the inputs y can be given by $x=\left\{x_{1},x_{2},\ldots ,x_{K}\right\}$, where $x_{k}\in \left\{0,1\right\}$. Here, the labels x are the encoded sequences corresponding to the inputs y.
- **Encoder-Hidden States:** The encoder has two hidden layers of size 5N. In the hidden layers, the hidden state is computed as $h_{j}^{\left(1\right)}=g\left(h_{j-1}^{\left(1\right)},y_{j}\right)$, where g(·) is a Relu activation function and $h_{j}^{\left(1\right)}$ is the *j*-th hidden unit of the first hidden layer. Then, $h_{j}^{\left(1\right)}$ is used to compute the second hidden layer, given by $h_{j}^{\left(2\right)}=g\left(h_{j}^{\left(1\right)},h_{j-1}^{\left(2\right)}\right)$. For the RNN cell, long short-term memory (LSTM) cell and gated recurrent unit (GRU) cell are widely used to avoid gradient vanishing as the network deepens. Moreover, GRU has less training parameters than LSTM [20]. Thus, we employ GRU cell in our work.
- **Encoder-Output:** The encoder has an output layer of size 1, which only outputs the result *c* of the final hidden state in the second hidden layer. To realize the single output of RNN-encoder, we set **return_sequences = False** in Keras. Note that the final output *c* contains all information of the received signal y so that the RNN-decoder can catch the information for further training.
- **Decoder-Input:***K* repeated *c* are fed into RNN-decoder as its inputs, which are given by $c=\left\{c_{1},c_{2},\ldots ,c_{K}\right\}$. Here, the $c_{i}\left(i=1,2,\ldots ,K\right)$ are the same.
- **Decoder-Hidden States:** The decoder has two hidden layers of size 5K. The working steps and its configure are the same as encoder-hidden states.
- **Decoder-Output:** The output layer is a fully-connected layer with the sigmoid activation function, given by $\sigma_{\mathrm{sigmoid}\;}\left(z\right)=1/\left(1+e^{-z}\right)$, with *z* being the input of the sigmoid function and $\sigma_{\mathrm{sigmoid}\;}\left(z\right)\in \left[0,1\right]$. Thus, the decoder outputs the final *K* soft estimates $\tilde{x}=\left\{{\tilde{x}}_{1},{\tilde{x}}_{2},\ldots ,{\tilde{x}}_{K}\right\}$ of transmitted data x. Here, ${\tilde{x}}_{k}\in \left(0,1\right)$, which indicates the probabilities of the RLL-decoded bit ${\hat{x}}_{i}$ being a “0” or a “1”. Therefore, we can output the final decoded sequence based on the rule (2).
- **Loss and Update:** Here, the mean square error (MSE) loss function is utilized to measure the uncertainty between the actual outputs $\tilde{x}$ and the desired outputs x, such that
  (6) $$L\left(x,\tilde{x}\right)=\frac{1}{K}\sum_{i=1}^{K}{\left(x_{i}-{\tilde{x}}_{i}\right)}^{2},$$
  where ${\tilde{x}}_{i}$ is the *i*-th output of decoder.
- **Training procedure:** To obtain the optimal parameter $\theta^{*}$ of the seq2seq decoder, a batch of the training dataset (i.e., a mini-batch) is randomly selected and fed into the decoder. As such, the loss function is computed using the outputs of decoder and the labels corresponding to the inputs. Later, the adaptive moment estimation (Adam) algorithm is executed to optimize the parameters of the NN. After repeated iterations with mini-batches, the parameters of NN converge and approach optimum values.

(2) *Testing Phase:* In this phase, the received signal y is first input to the seq2seq decoder. Then, the decoder outputs the soft estimates $\tilde{x}$ of transmitted data x. Finally, obtain the final RLL decoded sequence based on rule (2).

**Figure 3:**
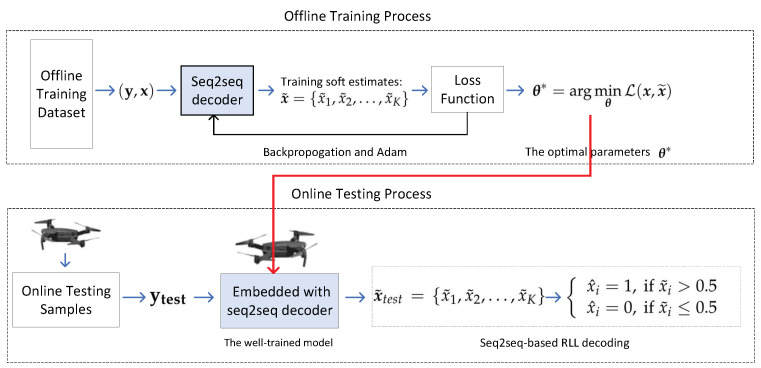
Offline training process and online decoding process of the proposed seq2seq-based decoding scheme.

As a final reminder, since the NN can efficiently execute in parallel and be implemented with low-precision data types on a graphical processing unit (GPU), field programmable gate array (FPGA), or application-specific integrated circuit (ASIC) [9], the well-trained decoder can be integrated into UAVs to decode RLL codes. A more detailed framework diagram of the proposed seq2seq-based decoding scheme is presented in Figure 3.

### 3.3. Dataset and Training Details

(1) *Data set:* To train the decoder, by transmitting the RLL-coded frames over the VLC model in Section 2.1 on different SNRs, we can generate a sufficient number of samples of received signals y and corresponding transmitted data (labels) x as the training and testing datasets. Further, the proposed seq2seq model can be trained over the whole codebook of RLL codes under various SNRs, since the codebook only contains up to dozens of codewords. This makes it easier to train a superior decoder.

(2) *Training SNR:* Note that the SNR is unknown and time-varying during the actual decoding phrase (testing phrase); hence, we trained an RLL decoder with generalization capability. That is, we trained the decoder at a particular SNR, and tested it on different SNRs. Naturally, the performance of the seq2seq decoder depends heavily on the SNR of the training samples. Next, section discusses the optimal training SNR for the seq2seq decoder. Our work employed the proposed method in [8] to obtain the optimal training SNR, and we define a new performance criterion, which is called the mean error to MAP (MEMAP) as follows:

(7) $$\mathrm{MEMAP}\left(s_{train}\right)=\frac{1}{T}\sum_{s_{\mathrm{test}}\in T}\left|{\mathrm{BER}}_{s2s}\left(s_{train},s_{test}\right)-{\mathrm{BER}}_{\mathrm{MAP}}\left(s_{test}\right)\right|,$$

where $s_{train}$ and $s_{test}$ represent the SNRs (defined by $E_{b}/N_{0}$) of training and testing sets, respectively. ${\mathrm{BER}}_{s2s}\left(s_{train},s_{test}\right)$ is the BER achieved by the seq2seq decoder trained at SNR $s_{train}$ and tested at SNR $s_{test}$. ${\mathrm{BER}}_{\mathrm{MAP}}\left(s_{test}\right)$ is the BER of MAP decoding of RLL codes on SNR $s_{test}$ and *T* denotes a set of different SNRs. The proposed criterion is able to evaluate how good a seq2seq decoder is and help us search the optimal training SNR, the details of which will be presented in the next section. Obviously, with MEMAP=0, the decoder achieves MAP decoding.

(3) *Network configuration:* Two RLL look-up tables are allowed to be employed simultaneously in the VLC standard to enhance the system throughput, and it is worth noting that our proposed seq2seq decoder is also adaptable to this task. If considering frame-by-frame transmission, i.e., the 4B6B codewords are transmitted and decoded one by one, the *N* of the decoder is set to 6 and *K* is set to 4. If we consider multi-frame transmission in one time slot, the *N* is set to a multiple of 6 and *K* is set to a multiple of 4, e.g., N=12,18, and K=8,12. For the setting of other hyperparameters in seq2seq decoder, we performed a lot of trials and determined relatively reasonable parameters that are summarized in Table 2. The performance of the decoder with this set parameters will be presented in the next section.

## 4. Results and Analysis

Extensive simulations were carried out to assess the performance of the seq2seq decoder. The VLC model described in Section 2.1 is the focus, and the SNR of the received signal is denoted as $E_{b}/N_{0}$, with $E_{b}$ being the energy per transmitted bit and $N_{0}$ being the variance of the Gaussian noise. For the performance comparison with seq2seq decoder, the look-up table and MAP decoding were adopted as the benchmark, which are the decoding methods used in the VLC standard and the ideal optimal decoding method, respectively.

### 4.1. Simulation Setup

For the two benchmark methods, we implemented them on Matlab R2019a. For the seq2seq decoder, it was built on Python with Keras libraries [21], and NVIDIA GTX 1070 Ti has been used to train the model. Further, the VLC model and corresponding dataset were generated on Python, and the testing data size was set to $10^{4}N$ bits per SNR for all subsequent simulations.

### 4.2. Optimal Training SNR

**Figure 4:**
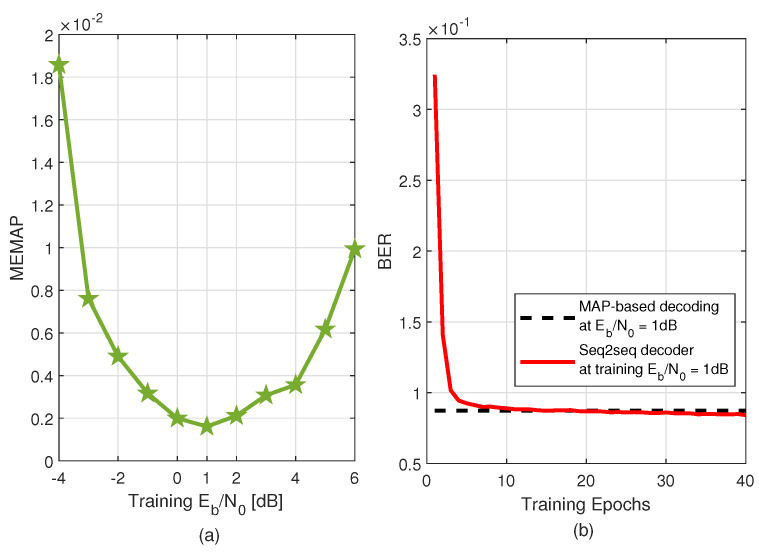
(**a**) MEMAP versus training $E_{b}/N_{0}$ over 4B6B RLL codes for a seq2seq decoder trained with 40 training epochs. (**b**) BERs of the seq2seq detector for each epoch during the training process at training $E_{b}/N_{0}=1\;\mathrm{dB}$.

Optimal training SNR was a prerequisite for our subsequent simulations; hence, this part discusses our results for searching for optimal training. Consider the frame-by-frame transmission case, i.e., N=6 and K=4, and we trained the seq2seq decoder with datasets of different training SNRs to observe the generated MEMAPs (proposed in Section 3.3). In our cases, we defined *T* in (6) as a set of 21 equally spaced SNRs points from −4dB to 6dB. It can be seen in Figure 4a that a training $E_{b}/N_{0}$ of 1dB leads to the lowest MEMAP, with MEMAP=0.0016. Hence, we set our training $E_{b}/N_{0}$ to 1dB for our subsequent simulations. To illustrate the learning process at training $E_{b}/N_{0}=1\;\mathrm{dB}$, we also plotted in Figure 4b the BER variations of the seq2seq decoder for each epoch during the training. We observe that the training BER of the decoder decreased as the epochs increased, and after several epochs, the BER converged and achieved the MAP-based decoding performance. This indicates that the proposed decoder can learn the MAP-decoding capability very well.

### 4.3. The BERs of the Seq2seq Decoder

To evaluate the performance of seq2seq decoder, we compare it with the traditional table look-up method. We also plotted the MAP decoding of 4B6B codes as the benchmark. That is, the received noisy signal y is decoded to the codeword c that minimizes the squared Euclidean distance $d_{E}^{2}\left(y,c\right)$ and naturally obtain the corresponding source word in terms of the 4B6B codebook.

**Figure 5:**
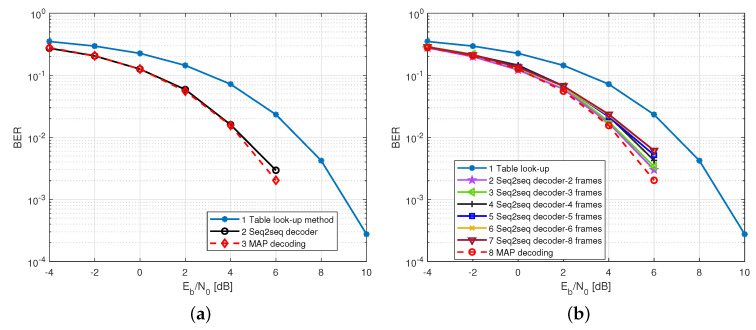
The BER performance of seq2seq decoder over 4B6B RLL codes. (**a**) Frame by frame transmission case; (**b**) multi-frame transmission case.

***(1) Frame-by-frame transmission***: Figure 5a shows the performance of the seq2seq decoder under the frame-by-frame transmission case. We can observe that the table look-up decoding method leads to the worst BERs performance, since it is a hard decision-based decoding method and is not able to make full use of channel information. Moreover, the seq2seq decoder had a notable performance gain of up to ∼2.5 dB at $\mathrm{BER}=10^{-2}$ compared to the conventional table look-up decoding. In addition, our seq2seq decoder can achieve a low BER performance that satisfies the MAP criterion.

***(2) Multi-frame transmission***: Figure 5b demonstrates the BERs of the seq2seq decoder under the case of processing multiple RLL-coded frames, where *N* is a multiple of six. It can be seen that the proposed decoder is fully capable of more than one frame of parallel decoding, and can reach eight frames of simultaneous decoding. The BERs almost achieved MAP-based decoding performance, which is able to largely enhance the throughput of the VLC systems. Note that such multi-frame decoding is achieved by a single decoding operation due to the parallel computing capability of the NN, whereas look-up table decoding requires multiple decoding operations to achieve multi-frame decoding. This, in a way, shows our method’s superiority over the traditional table look-up decoding method. However, with a further increase in the number of parallel frames, the performance of the seq2seq decoder is degraded. Therefore, we suggest that the number of frames for parallel decoding needs to be set in terms of the BER tolerance of the actual VLC application.

### 4.4. The BERs Comparison with Conventional RNN Decoder

To illustrate the necessity of RNN-decoder part in seq2seq decoder, we compare the performance of the seq2seq decoder with that of the conventional RNN decoder. That is, the decoder part of the seq2seq decoder was removed, we retained the encoder part with the same configure as before and we replaced the last layer of encoder with a fully connected layer of size *K*. We name this NN decoder the RNN decoder. Likewise, both NN-based decoders are evaluated under frame-by-frame transmission and multi-frame transmission cases.

**Figure 6:**
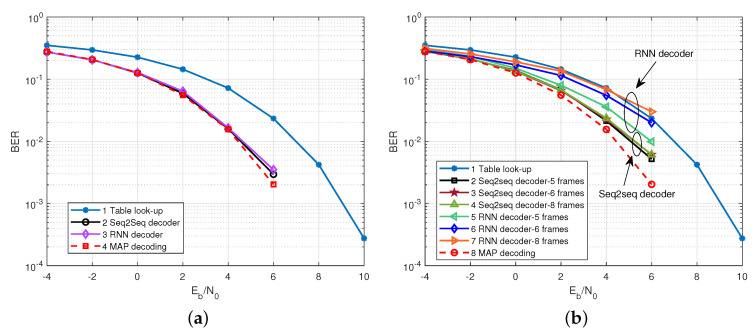
The BER performance comparison of seq2seq decoder and RNN decoder. (**a**) Frame by frame transmission case; (**b**) multi-frame transmission case.

Figure 6a,b shows the BER performances of two NN-based decoders under frame-by-frame transmission and multi-frame transmission. We can observe that in the frame-by-frame case, both decoders can approach the MAP decoding performance level and outperform the table look-up method. However, in the multi-frame transmission case, the performance of RNN decoder decreased as the number of transmitted frames increased (when the number of transmitted frames was 1–4, the performances of the two decoders were essentially the same, and therefore are not plotted in the figure). This indicates that the seq2seq decoder has a greater ability to transmit multiple frames compared to the RNN decoder, which enhances the throughput of the system, at the cost of having more training parameters than the RNN decoder.

### 4.5. Scalability

**Figure 7:**
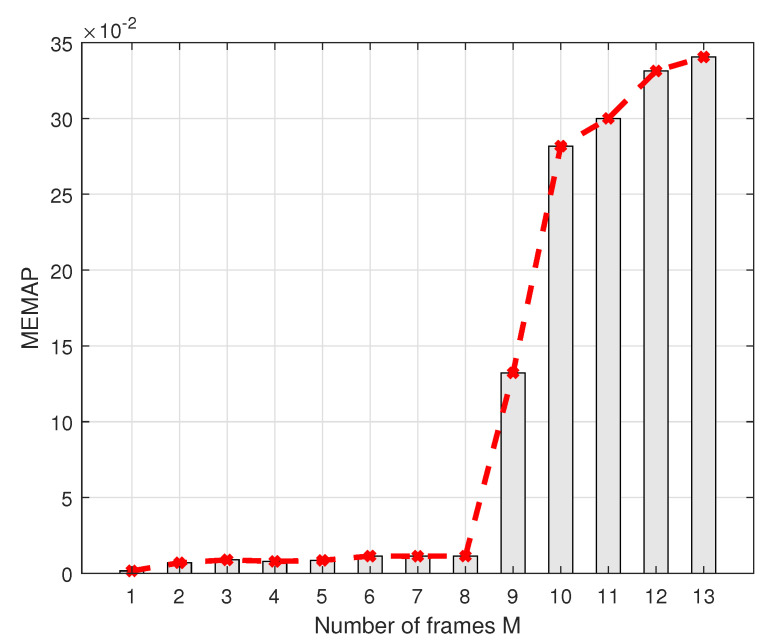
Scalability of seq2seq decoder indicated by MEMAP with different number of parallel decoding frames.

This section discusses the scalability of our decoder using the MEMAP criterion. Since our proposed criterion MEMAP can measure how good the seq2seq decoder is, the scalability of our decoder is discussed in Figure 7, which illustrates the variation of the MEMAP as the number of RLL-coded frames fed into the decoder increases. One can see that the MEMAP increases exponentially as the number of input frames increases. This is because, for a larger number of frames *M*, the decoder needs to distinguish more classes ($2^{M\cdot k}$, where *k* is the length of information bits for original 4B6B RLL codes), thereby degrading the generalization ability of the decoder due to the restricted learning abilities. Therefore, the number of transmitted frames is crucial and there exists a *saturation frame* for the RNN-based seq2seq decoder.

In Figure 7, we can further observe that M=8 is a point that requires our utmost attention. If the number of parallel frames exceeds 8, the MEMAP value will rise sharply. After a lot of trials, one fact that was proven is that our decoder was no longer capable of decoding properly and was thoroughly underfitting with more than eight frames, which indicates the *saturation frame* of our proposed decoder is frame 8 (assuming having a certain tolerance of performance loss). Thus, our proposed decoder is suitable for other RLL codes and shows strong prospects for practical applications of the VLC, and has ability to distinguish $2^{32}$ different codewords.

In addition, for a code with information bits k=32, the regular table look-up method with a codebook of $2^{32}$ source word-to-codeword mappings has been considered impractical, whereas our decoder can easily handle this task. Thereafter, our design of seq2seq decoder provides a reference to replace the look-up table decoding method for a code with fewer information bits.

## 5. Conclusions

In this paper, we adopted RNN technology to deal with the RLL decoding task for improving the reliability of the communication of UAVs embedded with VLC technology. Specifically, we transformed the RLL decoding task into a sequence-based problem that is suitable for the seq2seq model, and proposed a novel seq2seq decoder. Compared with the table look-up method, the well-behaved seq2seq decoder can obtain a performance gain. Meanwhile, it can achieve MAP bit-error-rate performance and enhance the throughput of a VLC system by decoding multiple RLL-coded frames simultaneously. In addition, due to the restricted learning abilities of the decoder, we found there exists a *saturation frame* and analyzed the upper limit of the scalability of our decoder, which provides a reference for practical VLC applications to select the number of parallel decoding frames.

## Figures and Tables

**Table 1:** The 4B6B codebook in VLC.

Source Word	Codeword	Source Word	Codeword
0000	001110	1000	011001
0001	001101	1001	011010
0010	010011	1010	011100
0011	010110	1011	110001
0100	010101	1100	110010
0101	100011	1101	101001
0110	100110	1110	101010
0111	100101	1111	101100

**Table 2:** The settings of seq2seq decoder.

Parameters	Seq2seq Decoder	
	RNN-Encoder	RNN-Decoder
Input Neurons	*N*	*K*
Hidden Layer Units	[5N, 5N]	[5N, 5N]
Output Neurons	1	*K*
Training sets	$4\times 10^{4}\;N$	
Batch size	100	
Training epochs	40	
Optimizer	Adam (with learning rate = 0.01)	
Initializer	Xavier uniform	
Loss Function	MSE	

## Data Availability

Not applicable.
